# Iodide of Starch

**Published:** 1897-11

**Authors:** 


IODIDE OF STARCH.—This is spoken of very highly by Dr. Pecus as presenting all the advantages of iodoform, and being much cheaper in price, and not having the peculiar, unpleasant odor of that drug: Tincture of iodine, 4 parts; starch, 6 parts. The iodine is added slowly to the starch, rubbed up and kept in a close vessel to prevent escape of the iodine.—*Revue Veterinaire.*

